# Sex-Related Differences in Pharmacological Response to CNS Drugs: A Narrative Review

**DOI:** 10.3390/jpm12060907

**Published:** 2022-05-31

**Authors:** Mirabela Romanescu, Valentina Buda, Adelina Lombrea, Minodora Andor, Ionut Ledeti, Maria Suciu, Corina Danciu, Cristina Adriana Dehelean, Liana Dehelean

**Affiliations:** 1 Faculty of Pharmacy, “Victor Babeş” University of Medicine and Pharmacy, 2 Eftimie Murgu Street, 300041 Timisoara, Romania; mirabela.romanescu@umft.ro (M.R.); adelina.lombrea@umft.ro (A.L.); ionut.ledeti@umft.ro (I.L.); suciu.maria@umft.ro (M.S.); corina.danciu@umft.ro (C.D.); cadehelean@umft.ro (C.A.D.); 2 Research Center for Pharmaco-Toxicological Evaluation, “Victor Babes” University of Medicine and Pharmacy, 2 Eftimie Murgu Square, 300041 Timisoara, Romania; 3 Faculty of Medicine, “Victor Babeş” University of Medicine and Pharmacy, 2 Eftimie Murgu Street, 300041 Timisoara, Romania; andor.minodora@umft.ro (M.A.); lianadeh@umft.ro (L.D.); 4 Advanced Instrumental Screening Center, “Victor Babes” University of Medicine and Pharmacy, 2 Eftimie Murgu Square, 300041 Timisoara, Romania

**Keywords:** CNS drugs, sex-related differences, pharmacological response, adverse drug reactions

## Abstract

In the last decades, both animal and human studies have neglected female subjects with the aim of evading a theorized intricacy of feminine hormonal status. However, clinical experience proves that pharmacological response may vary between the two sexes since pathophysiological dissimilarities between men and women significantly influence the pharmacokinetics and pharmacodynamics of drugs. Sex-related differences in central nervous system (CNS) medication are particularly challenging to assess due to the complexity of disease manifestation, drugs’ intricate mechanisms of action, and lack of trustworthy means of evaluating the clinical response to medication. Although many studies showed contrary results, it appears to be a general tendency towards a certain sex-related difference in each pharmacological class. Broadly, opioids seem to produce better analgesia in women especially when they are administered for a prolonged period of time. On the other hand, respiratory and gastrointestinal adverse drug reactions (ADRs) following morphine therapy are more prevalent among female patients. Regarding antidepressants, studies suggest that males might respond better to tricyclic antidepressants (TCAs), whereas females prefer selective serotonin reuptake inhibitors (SSRI), probably due to their tolerance to particular ADRs. In general, studies missed spotting any significant sex-related differences in the therapeutic effect of antiepileptic drugs (AED), but ADRs have sex variations in conjunction with sex hormones’ metabolism. On the subject of antipsychotic therapy, women appear to have a superior response to this pharmacological class, although there are also studies claiming the opposite. However, it seems that reported sex-related differences regarding ADRs are steadier: women are more at risk of developing various side effects, such as metabolic dysfunctions, cardiovascular disorders, and hyperprolactinemia. Taking all of the above into account, it seems that response to CNS drugs might be occasionally influenced by sex as a biological variable. Nonetheless, although for each pharmacological class, studies generally converge to a certain pattern, opposite outcomes are standing in the way of a clear consensus. Hence, the fact that so many studies are yielding conflicting results emphasizes once again the need to address sex-related differences in pharmacological response to drugs.

## 1. Introduction

Differences between the two sexes exist, and, from a medical point of view, they have a significant impact on prevalence, incidence, and severity of a wide range of diseases and conditions. Accordingly, physiologic differences between the two sexes affect drug activity with dissimilarities within pharmacokinetics, pharmacodynamics, and pharmacotoxicity. However, most drugs are prescribed to women and men at the same dose, although therapeutic effectiveness varies (Table 1) [1]. In research, women and non-human female mammals have often been underrepresented, especially in previous decades. The reasoning behind it is the assumption that results from males readily apply to females, or the concern that hormonal cycles negatively influence the homogeneity of study populations and complicate experimental designs [2]. Moreover, the risk of ADRs such as teratogenicity or toxicity may outweigh other considerations, and, thus, females of child-bearing potential, pregnant, or breastfeeding are sometimes advised by healthcare professionals against enrolling in such studies (Table 2) [3].

Pharmacokinetics in women is influenced by several factors, such as: lower body weight, higher percentage of body fat, slower gastrointestinal motility, higher gastric pH, decreased intestinal enzymatic activity, and slower glomerular filtration rate [4]. With regard to medication, drugs in women usually have a larger volume of distribution, and the free fraction is also increased. Female sex hormones alter hepatic enzyme activity, which can result in decreased elimination and accumulation for some drugs. However, the way estrogen and progesterone affect pharmacokinetics of drugs is hard to predict and assess, with studies yielding conflicting results [5]. Differences in pharmacodynamics occur when the same plasma concentration of a drug does not cause the same pharmacological response between the sexes. Unlike pharmacokinetic differences, pharmacodynamic disparities are more difficult to assess, as pharmacological effects are not easily measurable [6]. However, there are certain examples in the literature where such differences are obvious. To name a few, women are more likely to experience QT interval prolongation following therapy, while males show a greater sensitivity to propofol’s anesthetic effect [7]. Another example would be that verapamil has a lower bioavailability and increased clearance in male patients compared to females [8]. Regarding pharmacotoxicology, women are significantly more likely to be hospitalized secondary to an ADR, as they have a nearly two-fold greater risk than men for exhibiting side effects across many drug classes [9]. For instance, women appear to be at a higher risk for ADRs following treatment with thyroid hormones, phychoanaleptics, and TNF-α inhibitors [10]. Additionally, women are more at risk of admission using thiazide diuretics causing hypokalemia and anticoagulants causing rectal bleeding, whereas males have higher rates of hematuria and subdural hemorrhage following treatment with anticoagulants [11].

The purpose of this narrative review is to explore to what extent the response to CNS medication is influenced by sex as a biological variable in: drug delivery, pharmacokinetic response, drug efficacy, and ADRs. The aim is to identify and summarize how the observed sex-related differences are corelated with: a disease’s pathophysiology, epidemiology, drugs’ underlying mechanisms of action, a patient’s habits/medication, or any other interfering factor.

## 2. Materials and Methods

### 2.1. Literature Search Strategy

From January 2021 to June 2021, we searched five electronic databases (PubMED, Web of Science, Elsevier’s Scopus, Google Scholar, and PsychINFO) for papers analyzing treatment response to various CNS diseases. We intended to identify relevant articles from 1980 to 2021 and limited our analysis to articles written in English.

Searches were conducted separately in each database, and the records were exported to citation software after removing the duplicates. Search terms were utilized in several combinations, taking into account the possibility of encountering various suffixes (including plural terminations), hereby represented by the * character. The primary search terms were: analgesia, opioid *, depression OR depressive, antidepressant *, epilepsy, anticonvulsant * OR antiepileptic *, psychosis OR schizophrenia, antipsychotic *, sex OR gender difference *, male * OR men, female * OR women (e.g., (depress * OR antidepressant *) AND (gender OR sex) AND (difference * or dissimilarity * OR distinction)). For each distinct pharmacological class, a manual search was performed on the same electronic databases in order to look into additional distinctive keywords and broaden the results range (*e.g., morphine, ISRSs, atypical antipsychotics, etc.*).

Afterwards, we searched reference lists of identified articles to ensure the capture of significant literature missed during the primary search. Then, we checked relevant review papers for supplementary references. Lastly, with the aim of elucidating the various results obtained in studies, for each of the diseases tackled in the present review, we consulted articles approaching sex-related differences in epidemiology, pathophysiology, prognostic, and co-morbidities.

### 2.2. Selection Criteria

This narrative review analyzed articles that met the following inclusion criteria:

- Human studies (clinical trials, experimental studies, case reports, reviews, meta-analysis);
- Studies in adult patients (subjects > 18 years);
- Studies that investigated sex differences.

When any of these criteria were not met, papers were eliminated from the analysis. We also excluded studies that:

- Investigated animal populations;
- Failed to analyze outcomes and differentiate between men and women;
- Were methodologically flawed;
- Provided insufficient details or irrelevant outcomes.

### 2.3. Selection Process

**Figure 1:**
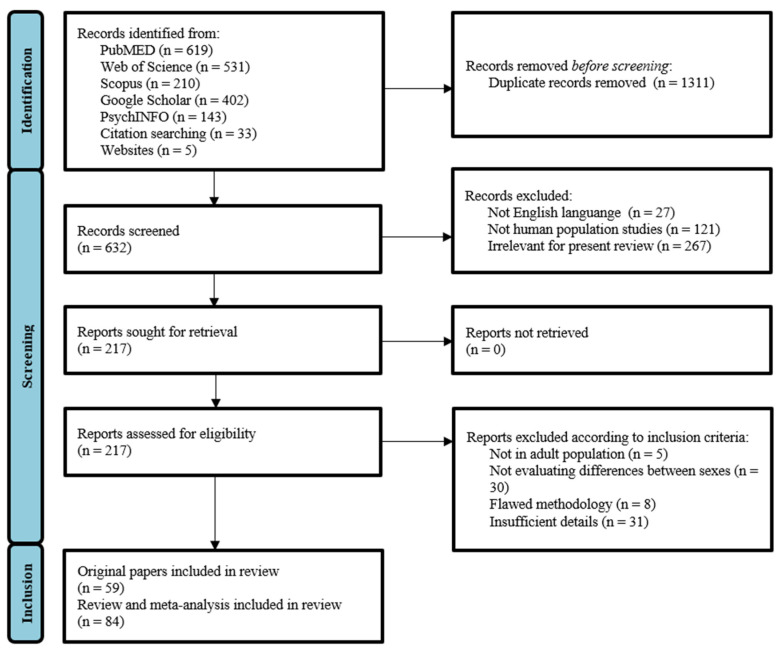
Flow chart of studies identified and selected for this review.

Electronic database searches, alongside supplemental searching strategies, yielded a total number of 1943 records (Figure 1). After removing the duplicates, 632 studies remained for further examination. A first selection was performed based on titles and short abstract screening, narrowing down the range to 217 records. All articles that examined sex differences were retained, even if this was not the primary focus of the study, as long as the search terms were mentioned in the publication title, abstract, or subject headings. Then, two reviewers (V.B. and L.D.) independently restricted the analysis to those articles that met the inclusion criteria. The reviewers reached consensus and performed the final selection procedure including inspection of overall study quality and risk of bias. This resulted in a total number of 143 eligible articles, including 59 original papers and 84 reviews and meta-analyses. All remaining papers were read in full.

### 2.4. Data Extraction

The results of the pertinent articles were closely evaluated by two reviewers (V.B. and L.D.). Each selected paper was assessed, and the following variables were extracted: study design, number of patients, disease, medication under study, dose, duration of treatment, response to treatment (efficacy and/or ADRs). Any disagreements or queries during the writing of the article were settled by contacting the two reviewers (V.B. and L.D.) and reaching a consensus.

## 3. Opioids

It is well known that pain sensitivity varies among individuals; therefore, assessing and treating postoperative pain requires a personalized approach, making it difficult to follow protocols strictly. An effective analgesia needs to take into account various factors, such as weight, height, age, body mass index, sex, type of surgery, surgery site, preoperative pain, and medication [12]. It is established that the majority of chronic pain syndromes occur more often in women (chronic fatigue syndrome, fibromyalgia, interstitial cystitis, temporomandibular disorder, headache, migraine, low back pain, neck pain, and osteoarthritis) [13]. Likewise, studies indicate a greater pain prevalence among women and determined that women seem to show greater sensitivity to the majority of experimentally induced pain methods [14,15,16]. However, the differences in pain perception observed within most studies may not be statistically significant and are not always consistent, as suggested by a series of review papers [17,18,19,20]. Pain perception seems to be linked to sex hormones, since testosterone was found to decrease pain sensitivity; thus, a low testosterone state is incriminated in a wide range of chronic pain conditions. Nevertheless, female hormones have both pro- and antinociceptive properties, making the effects of estrogen and progesterone on pain more difficult to evaluate [21]. Regarding opioid addiction, it is established that the desire for opioids is considerably higher among women, and they are at an increased risk of abusing opioids through initial prescription painkiller use [22].

Most pain-related animal studies only include male subjects; few are focused on females, and just a small number are explicitly designed to test for sex differences [23]. Animal studies reveal that, generally, opioid analgesia is more effective in males compared with females. It is known that adult female rodents have a lower percentage of body fat than males, whereas the situation is opposite in humans. These sex-related differences may affect the distribution of highly lipophilic drugs, having a substantial impact on drugs’ potency, efficacy, and duration of action [23,24,25]. However, results in human subjects are not as consistent as those in rodents.

Although recent years were marked with significantly increased research regarding sex differences in pain, studies on gender differences in opioids concluded a mixture of different results. Most papers focus on μ agonists, especially morphine, as a main treatment for pain alleviation, since it is perhaps the most clinically significant opioid [26]. Existing data regarding sex differences in response to morphine are highly inconsistent, and a general assumption is difficult to make. It appears that discrepancies in the sex-related response to morphine analgesia might virtually depend on the pain model and/or drug dose/regimen used.

To begin with, some clinical studies investigating the difference in the postoperative morphine requirements determined that male opioid consumption was higher than the one observed in females through patient-controlled analgesia (PCA) [12,27,28]. This means that women self-administer significantly less morphine than men [29]. In addition, further research showed that women experience greater morphine potency, as well as a slower speed of onset and offset of the analgesic effect. However, no sex differences in plasma concentrations of morphine and its major metabolites (morphine-3 and 6-glucuronide) were observed. Those findings suggest that gender differences in opioid analgesia are not related to morphine’s pharmacokinetics [25,30]. On the other hand, when analyzing the immediate postoperative analgesia, following intravenous titration of morphine, it appears that women require a higher dosage than men [31,32]. This might be explained by the slower onset of morphine in women, who experience later analgesia. Contrarywise, other research papers reported similar analgesic effects of morphine in both sexes. These studies used experimental pain models and evaluated the response to intramuscular morphine by measuring its plasma levels as well as subjective experience, performance, and physiological effects [33,34]. However, the results on elderly patients appear steadier, with no significant differences in the analgesic effect of morphine being observed in most studies [31,35].

Regarding ADRs, there is considerable, as well as consistent, evidence that sex influences the intensity and frequency of morphine’s side effects. Findings indicate that females have a substantially higher risk of developing nausea and vomiting than men following opioid analgesia [36]. These observations might be related to the higher frequency of post-operative nausea and vomiting among women than men [37,38]. However, the same results were also obtained in a study of narcotic-induced emesis in the emergency department, which strengthens the initial conjecture [39]. Furthermore, additional studies reported greater morphine-induced respiratory depression in females than in males [40,41] as well as an increased feeling of disorientation and sluggishness [42]. In addition, preliminary results suggest some cardiovascular differences between the two sexes following IV morphine administration: women experienced a lower heart rate, but only men developed hypertension and had an attenuated cardiovascular response to ischemic pain. However, the observed differences were small, and only one low morphine dose was tested; therefore, further investigations are needed [34].

Leaving morphine aside and analyzing opioids as a whole, the same conclusion can be drawn—distinct investigations reveal various results. It appears that earlier studies tend to deduce that opioids are better analgesics for females [43,44,45]. However, it is hypothesized that this may happen because older studies did not correct for the body weight differences between the two sexes [46]. For instance, the mixed µ-k-opioid agonist-antagonist nalbuphine, butorphanol, and pentazocine produced significantly better postoperative analgesia in women than in men [47]. In contrast, other studies using experimental pain models showed that pentazocine produced analgesia of similar magnitude in men and women [48,49]. Regarding µ-opioids, a similar analgesic response in the two sexes is achieved after the administration of alfentanil, as well as morphine’s active metabolite, morphine-6-glucuronide [50,51]. Considering PCA studies on µ-opioids, it generally appears that opioid consumption is higher in men than in women. However, most studies actually assess opioid consumption rather than pain relief. Therefore, PCA poses problems in terms of reliability, as it can be influenced by other factors than just postoperative pain, such as: expectations, baseline pain sensitivity, fear of addiction, and the occurrence of unpleasant ARDs, such as nausea and vomiting [52].

The general verdict regarding the diverse outcomes of studies is that results may be influenced by procedural and subject variables. Broadly, opioids seem to produce better analgesia in women, especially when they are administered for a few days, as the onset of action is delayed in this category of patients. However, the various responses to pharmacological pain interventions appear inconsistent and dependent on treatment type, genotype, gonadal steroid hormone state of subjects, and characteristics of the pain and the provider [14,16,26,53].

## 4. Antidepressants

Epidemiological studies demonstrate that in children the rates of depression are equivalent between the sexes, but adolescence makes women more than twice as likely to suffer from depression than males. Females also experience more atypical symptoms than men, show greater severity, earlier age of onset, and increased duration of depressive episodes [54,55]. Atypical symptoms are often associated with greater depression severity and more overall comorbidity, and because of that women are also more likely to report comorbid anxiety [56].

Both the pharmacokinetics and pharmacodynamics of antidepressants seem to be affected by physiological and molecular differences between the two sexes. The majority of antidepressants are weak bases, so they are more effectively absorbed from women’s gastrointestinal tract since females secrete less gastric acid and have a more basic environment [57].

In terms of physiological differences, two studies highlighted that patients with lower body weight, generally women, responded preferentially to the SSRI fluoxetine [58,59]. Additionally, the magnitude of the effect of body weight on antidepressant activity seems to depend on gender. A study of the response to a number of SSRIs reported differences between sexes related to body mass index. Specifically, obese men failed to respond to any of the tested SSRIs better than a placebo, while obese women showed superior response when compared to a placebo [60]. Moreover, antidepressants are mostly strongly lipophilic; therefore, their volume of distribution is higher in women than in men. This might explain why trazodone and bupropion have an increased half-life in women with this effect being further highlighted in elderly females [61].

Plasmatic level of tryptophan is more reduced in females than in males, which leads to a deficiency in serotonin and, consequently, to depression [62,63]. It is suggested that estrogen can affect the production of serotonin as well as the expression and binding of serotonin to transporter receptors within its pathway, processes that have a direct impact on SSRIs’ efficacy [56]. This might explain why women in their reproductive period are more responsive to SSRIs. Moreover, it appears that female patients produce less cortisol and more tryptophan when exposed to SSRIs [64].

Moving on to proteins and enzymatic activity, it looks as though women may have a lower activity of P-gp, a drug-efflux pump found in the gut and brain, which lowers the uptake of substances. Animal studies showed that the inhibition of P-gp may amplify the uptake of various antidepressants, such as nortriptyline, amitriptyline doxepin, venlafaxine, citalopram, and paroxetine [65,66]. A review analyzing gender differences in antidepressants found that plasma levels of TCAs imipramine, nortriptyline, and desipramine are reportedly higher in women, consistent with lower P-gp activity at absorption and lower activity of CYP1A2 and CYP2C19 enzymes. Therefore, antidepressants may have higher serum levels in women due to decreased expression and inhibition of many of the phase I enzymes necessary for the metabolism of these drugs [56]. In addition, some SSRIs, such as fluoxetine, fluvoxamine, or, to a lesser extent, paroxetine and sertraline, are known to be able to inhibit certain CYP enzymes involved in the metabolism of co-administered drugs [67]. For example, it is not uncommon to see benzodiazepines prescribed alongside SSRIs for a variety of reasons: (1) reduction in SSRI-induced anxiety which occurs early in the course of therapy; (2) improvement in adherence to antidepressant therapy, (3) control of comorbid anxiety associated with atypical symptoms. As stated previously, females are more likely to associate anxiety and atypical symptoms; therefore, they could benefit more from this association [68,69]. Fluoxetine was reported to decrease the metabolic clearance of benzodiazepines such as diazepam and alprazolam through inhibition of CYP-2C19 (diazepam) and CYP-3A4 (diazepam, alprazolam). As a consequence, fluoxetine might increase benzodiazepines’ plasmatic concentration with the risk of exacerbating their effects [70,71]. The same can be said of fluvoxamine, which was shown to impair the elimination of alprazolam and diazepam by interfering with the same CYP enzymes [72].

Recent reviews and clinical studies reported that females experience an increased efficacy of pro-serotoninergic antidepressants, while men respond better to pro-noradrenergic drugs [55,56,73,74,75]. Generally, women are more likely to respond favorably to fluoxetine and citalopram, whereas men prefer the tetracyclic antidepressant maprotiline [76,77]. Female patients are also more likely to respond favorably to sertraline than to imipramine, whereas the opposite might be said for men, although to a lesser extent [78,79]. Other papers suggested that women showed a superior response to SSRIs than to serotonin and norepinephrine reuptake inhibitors (SNRIs) such as venlafaxine. They also responded better responses to citalopram than to the selective noradrenergic reuptake inhibitor reboxetine [75,80]. This can happen because women have the tendency to display more somatic symptoms associated with atypical depression, therefore responding preferentially to SSRIs [81]. Further research revealed that younger women generally have a better response to SSRIs than women aged 50+ years [78,79,82]. Atypical depression symptoms can be also cured using monoamine oxidase inhibitors, which were reported to produce a larger response in women than in men, although other findings suggest no sex response difference within the class [83,84]. On the other hand, there are also studies that report no difference in efficacy in men and women for antidepressants such as fluoxetine, clomipramine, citalopram, paroxetine, and moclobemide [84,85,86]. Furthermore, there are not many studies concerning newer non-SSRI antidepressants, but few reported that there was not any sex difference in response to venlafaxine, bupropion, or sertraline [87].

Another aspect worth mentioning is the use of augmentation therapy—drugs that have other primary indications that are thought to enhance the effect of antidepressants. For example, folic acid supplementation enhances the effect of fluoxetine in female patients [88], while the triiodothyronine hormone accelerates the response to TCA, and this effect is more pronounced in women [89]. As previously stated, females also benefit more from the association with benzodiazepines [68]. More than that, hormone replacement therapy was found to eliminate a poor SSRI response in older women; therefore, estradiol might augment SSRIs’ effectiveness [82].

With regard to adverse drug reactions, women seem to have a lower tolerance for imipramine than sertraline, imipramine being responsible for increased instances of constipation, sweating, dry mouth, and tremor. For this reason, women were almost three times more likely to cease treatment with imipramine than sertraline, whereas men showed similar drop-out rates [78,79]. The fact that women are more likely to experience adverse drug reactions to tricyclic antidepressants is probably linked to pharmacokinetic studies which concluded that women have higher plasma levels of these drugs than men. Another hypothesis is that women and men have a different tolerance of particular adverse reactions, with the anticholinergic effects (dry mouth, constipation, sedation, sweating, and tremor) associated with TCAs possibly being more acceptable to men than the ADRs associated with SSRIs [56]. The activation of the 5-HT2 receptors impairs all stages of the sexual response. Therefore, although SSRIs induce less ADRs than TCAs, they deteriorate the sexual function precisely through: impairment in desire and arousal, inhibition of orgasm, delayed ejaculation, and male impotence [90,91,92]. Apart from inhibiting the reuptake of noradrenaline and serotonin, TCAs are also known to antagonize postsynaptic α1-adrenoceptors, histamine (H1) receptors, muscarinic cholinergic receptors, and serotonin (5-HT2) receptors, resulting in a series of ADRs including sedation [93]. Because of its sedating effects, this class of antidepressants was used in the past as the first-choice agent in the treatment of comorbid anxiety or insomnia, which, as mentioned above, is more frequent in females [94,95].

As previously stated, most studies show that male patients respond better to TCAs, whereas females prefer SSRIs. This tendency seems to be especially dictated by the tolerance to particular ADRs, with anticholinergic effects being less desirable in women, and sexual dysfunction being displeasing to men. However, as with opioids, conflicting outcomes restrict the freedom of drawing a generally valid conclusion.

## 5. Anticonvulsants

Epilepsy—one of the biggest causes of chronic neurological morbidity—has a specific sex incidence with males being relatively more susceptible to seizures than females [96,97,98]. Although women are not as frequently affected by epilepsy as men, they experience more complex and refractory seizure syndromes. This might be explained by the effects that hormones have on seizures, with numerous women stating that their seizure manifestation goes through different stages at puberty, the menstrual cycle, and menopause [96]. Closely linked to menstrual cycle periodicity is hormonal contraception, which seems not only to interact with anticonvulsants, but also to exacerbate the seizures. While estrogen seems to exert an excitatory effect by increasing glutamate and decreasing GABA, progesterone appears to be doing the opposite, which leads to the conclusion that the first hormone promotes seizures, whereas the other one reduces their frequency [98,99,100].

There are not many reports on sex differences in the anti-seizure effect of anticonvulsants. The results reported by clinical trials either do not address gender dissimilarities, or findings are not significant. Sex-related differences in the pharmacokinetics of antiepileptic drugs (AEDs) are reported occasionally, but they lack in consistency and clinical significance. Although it is expected that estrogens accelerate the rate at which drugs are being glucuronidated, studies hardly show any relevant changes in the plasma concentration of anticonvulsants in women compared to men [101,102]. However, despite the fact that variation in endogenous hormones during a regular menstrual cycle has no significant impact on serum concentrations of AEDs, a study conducted in 2009 revealed that women who take oral contraceptives have a substantially reduced concentration of lamotrigine and valproic acid in the serum [101,103].These findings suggest that drug interactions may occur with medications that are used specifically in one of the two sexes. Moreover, additional research papers indicate that certain AEDs, mostly enzyme inducing drugs (carbamazepine, phenytoin, phenobarbital, primidone, and also lamotrigine, topiramate, felbamate, oxcarbazepine, eslicarbazepine acetate, and rufinamide) reduce the contraceptive efficacy of: the combined oral contraceptive pill, combined contraceptive patch, vaginal ring, progestogen-only pill (minipill), progestogen implant, and postcoital contraceptives [104,105]. AEDs’ serum concentration can be affected by another medication with gender-specific indications—tamoxifen—a drug used in the treatment of breast cancer. This molecule seems to increase plasma levels of phenytoin and possibly to cause clinical signs of phenytoin toxicity [106]. Another drug interaction relevant to the male gender is tadalafil which, according to the Summary of Product Characteristics, seems to be affected by CYP3A4 inducers, such as phenobarbital, phenytoin, and carbamazepine [107].

One recent review analyzing the well-known pharmacokinetic changes during pregnancy revealed important drops in the serum concentration of several anticonvulsants. The most prominent effect appears to occur with lamotrigine, which can be explained by its elimination route through glucuronidation [108,109]. Oxcarbazepine, a prodrug following the same metabolic route as lamotrigine, behaves in a similar manner, having plasma levels reduced by 30–40% [110]. Antiepileptic drugs cleared by other routes seem to be as well considerably affected during the gestation period. As stated by Tomson et al., levetiracetam plasma levels declined during the first two trimesters and dropped even more sharply in the third one compared to baseline [111]. Following the same pattern, pregnant women experience decreased levels of phenytoin, phenobarbital, topiramate, and valproate, while other drugs such as carbamazepine seem to be inconsistently affected by pregnancy [108].

Certain ADRs of AEDs seem to be considerably influenced by gender, most of them consisting of changes within sex hormones’ metabolism [101]. In women, AEDs such as carbamazepine, phenytoin, and phenobarbital appear to be responsible for a variety of sex hormones’ abnormalities, including low levels of total serum testosterone, free androgen index, dehydroepiandrosterone sulfate, and estradiol, alongside increased levels of sex-hormone-binding-globulin. These drugs produced sexual dysfunction and low arousal scores as well as a concerning health problem for postmenopausal women—alteration in bone metabolism [112,113]. In males, enzyme inducing anticonvulsants determined a drop in androgen levels and a series of sexual dysfunctions, such as reduced fertility, reduced sperm counts, and morphological sperm abnormalities [113,114,115]. Valproic acid, one of the non-enzyme inducing AEDs, was incriminated in producing gender-related side effects, especially among women, increasing the incidence of polycystic ovary syndrome, hyperinsulinism, hyperandrogenism, hypothalamic amenorrhea, and functional hyperprolactinemia apart from the acknowledged effects on offspring [116].

Generally, studies did not find any significant sex-related difference in response to AEDs. The results reported by clinical trials either do not address gender dissimilarities, or findings are not statistically significant. Anticonvulsants can lead to various changes within sex hormones’ metabolism, which is particularly why many of them determine such ADRs. However, since AEDs have a multitude of mechanisms of action, a universal conclusion over sex-related disparities cannot be drawn, with different drugs exhibiting a different ADRs’ profile.

## 6. Antipsychotics

There are a few sex differences that affect drug response in neuro-psychiatric disorders, such as schizophrenia. First of all, women receive a later diagnosis since the onset of the disease is delayed [117]. Deficit symptoms are less prevalent in women with schizophrenia, and they experience less severe symptoms, fewer hospitalizations, and shorter admissions [118,119]. Therapeutic adherence is higher in women, and they have better outcomes due to lifestyle, social support, the advantage of later onset, and relative hormonal protection. Moreover, associated pathologies are more common among females, such as pain conditions, allergies, mood problems, sleep disturbances, endocrine disturbances, eating disorders, and personality disorders. Therefore, they require additional medication and are more prone to drug interactions. Conversely, men smoke more and are more likely to abuse multiple street drugs and alcohol. It is established that smoking status strongly affects the metabolism of certain drugs. Therefore, all the factors mentioned above lead to a poorer prognosis [120,121].

On the subject of antipsychotics’ pharmacokinetics, the volume of distribution of these lipophilic drugs is greater in women than in men, leading to prolonged half-life and to accumulation over time [120]. Women eliminate antipsychotics slower than do men due to their decreased glomerular filtration rate, renal tubular secretion, and reabsorption, all contributing to drug accumulation within the body [122]. For instance, the summary of product characteristics of olanzapine shows a different elimination profile in female versus male subjects, with women having a prolonged mean elimination half-life (36.7 versus 32.3 h) and a reduced clearance [123].

**Table 1:** Sex-related differences in therapeutic effectiveness.

Pharmacological Class/Drug	Effectiveness		Comments and Conclusion	Reference
	Results	Consistency		
Opioids	F > M	C	Mixed µ-k-opioid agonist-antagonists and pure µ-agonists appear to be slightly more effective in women judging by the consumption of opioids in the two sexes.	[43,44,45,46,47,48,49,50,51]
Morphine	F > M	B	The majority of studies indicate that immediate postoperative analgesia is less effective in women since they experience a slower speed of onset. Contrarywise, PCA shows that female patients self-administer significantly less morphine than males.	[12,25,27,28,29,30,31,32,33,34,35]
TCAs	M > F	C	Males report an increased efficacy of pro-noradrenergic drugs probably due to their lower tolerance to sexual dysfunctions associated with SSRIs.	[55,56,73,74,75,76,77,78,79]
SSRIs	F > M	C	Women respond better to pro-serotoninergic drugs because anticholinergic ADRs associated with TCAs might be less desirable to these patients.	[55,56,73,74,75,76,77,78,79]
Anticonvulsants	M = F	B	There are few studies available, which either do not address gender dissimilarities, or their findings are not statistically significant.	[101,102]
Antipsychotics	F > M	C	Antipsychotic response seems to be higher in females, but this may simply indicate that, compared to males, they are at an earlier stage of illness.	[124,125,126,127,128,129,130]

Legend: B—Indicated by many studies, although there are some stating the opposite; C—conflicting results, but there is a tendency towards the indicated direction; F—Females; M—Males; PCA—patient-controlled analgesia.

The clinical dissimilarities between antipsychotic drugs are mostly in the areas of safety and tolerability [124]. As stated by a systematic review of gender differences in schizophrenia treatment restricted to randomized controlled trials and meta-analyses, there is evidence that points to an inferior antipsychotic response in men, who require a higher dosage in order to achieve an equivalent drop in psychotic symptoms [125]. However, when comparing male/female results in drug trials, it is essential to take into account that response to antipsychotics is nearly always better during early episodes of schizophrenia than it is after multiple episodes. Therefore, women’s superior response to therapy may simply indicate the fact that, compared to men, they are at an earlier stage of illness [126].

Neuroleptic-naive women show a better response to antipsychotic treatment than neuroleptic-naive men. Moreover, males have a reduced likelihood of response to antipsychotic treatment at 1 year compared with females; yet, there are no sex differences in risk of relapse [122]. Gender appears to be a significant predictor of response to antipsychotic treatment with women responding better and requiring lower doses than men [127,128]. One study following long-term antipsychotic treatment response and serum levels suggests that males require twice the dose of antipsychotic compared with females [129]. In contrast, another paper showed that oral haloperidol, risperidone, and olanzapine are all equally effective, but men respond better to acute treatment than women do, both during the initial 2 h period as well as over the 5-day course [130].

Schizophrenic patients are reported to have a higher prevalence of smoking than the general population. Certain antipsychotics, namely clozapine and olanzapine, are highly influenced by smoke constituents in cigarettes. Polycyclic aromatic hydrocarbons are inducers of the CYP1A2 hepatic enzyme [131]. The main metabolic pathway for clozapine and olanzapine is N-demethylation, a reaction catalyzed by the cytochrome isoenzyme in question. Thus, enzyme induction results in accelerated metabolism of these two drugs, lower plasma levels, and, thereupon, lower efficacy and therapy refractoriness [132]. On the other hand, substantial reduction in the number of smoked cigarettes or total smoking cessation results in increased plasma levels of olanzapine/clozapine and can have deleterious consequences. Hence, patients undergoing treatment who are advised by health-care professionals to quit smoking or who are forced to do so upon hospital admission may be at a high risk of developing serious ADRs [133]. As previously stated, men are more prone to tobacco consumption than women, so male patients should be monitored even more strictly especially regarding their smoking cessation plans [120].

**Table 2:** Sex-related differences in ADRs.

Pharmacological Class/Drug	ADRs’ Frequency/Intensity			Comments and Conclusion	Reference
	Results	Consistency	Type of ADRs		
Morphine	F > M	A	Nausea, vomiting, respiratory depression	Gastrointestinal and respiratory ADRs are considerably more frequent in women. There are hints that cardiovascular ADRs are also influenced by sex, but the available data are scarce.	[36,37,38,39,40,41,42]
TCAs	F > M	B	Dry mouth, constipation, sedation, sweating, and tremor	Pharmacokinetic studies revealed that women have higher plasma levels of TCAs than men, therefore being more sensitive to side effects.	[56,78,79]
SSRIs	M > F	B	Sexual dysfunction	SSRIs deteriorate the sexual function precisely through: impairment in desire and arousal, inhibition of orgasm, delayed ejaculation, and male impotence.	[90,91,92]
Anticonvulsants	-	I	Sex-hormone-related ADRs	Generally, AEDs can lead to changes within sex hormones’ metabolism. However, since these drugs have a multitude of mechanisms of action, a general conclusion over sex-related differences cannot be drawn.	[101,112,113,114,115,116]
Valproic acid	F > M	B	Polycystic ovary syndrome, hyperinsulinism, hyperandrogenism, hypothalamic amenorrhea	Valproic acid has been incriminated in producing gender-related side effects, especially among women, increasing the incidence of the mentioned ADRs, apart from the acknowledged effects on offsprings.	[116]
CarbamazepinePhenytoinPhenobarbital	F > M	B	Alteration in bone metabolism	They increase the levels of sex-hormone-binding-globulin and decrease the levels of total serum testosterone, free androgen index, dehydroepiandosterone sulfate, and estradiol.	[112,113]
Antipsychotics	F > M	B	metabolic dysfunctions, cardiovascular disorders, hyperprolactinemia	Females exhibit lower fasting plasma glucose levels, elevated waist circumference and waist-to-hip ratio, prolonged QTc interval, and reduced bone density due to hyperprolactinemia.	[124,134,135,136,137]
	M > F	C	acute dystonic reactions, tardive dystonia, akathisia	Males are generally more prone to developing extrapyramidal side effects.	[122]
	M = F	B	Sexual dysfunction	ADRs are due to dopaminergic antagonists (females) or drugs having α1-antiadrenergic/anticholinergic properties (males).	[138,139,140,141,142,143]

Legend: A—Indicated by the majority of studies; B—Indicated by many studies, although there are some stating the opposite; C—conflicting results, but there is a tendency towards the indicated direction; F—Females; M—Males; I—Inconsistent Results.

Regarding adverse drug reactions (ADRs), females suffering from schizophrenia seem to be diagnosed with metabolic diseases at higher rates than males. A thorough study divided antipsychotic drugs in 3 groups according to their metabolic risk: high—clozapine and olanzapine; moderate—quetiapine, risperidone, paliperidone, and iloperidone; low—all other antipsychotics. According to the results, women had lower fasting plasma glucose levels but an elevated waist circumference and waist-to-hip ratio compared to men. Surprisingly, glucose levels were elevated even within the low-risk group, although medication in this category is not associated with this kind of side effect. Additionally, men have higher diastolic and systolic blood pressure than women, but values seen in both sexes fell within the normal range. On the other hand, although men have a lower total cholesterol, LDL, and HDL, both males and females are at risk for medication-specific lipid dysregulation [134,135,136,137].

Schizophrenic patients have a reduced life expectancy that can be assigned to higher rates of cardiovascular disease [124]. For example, women following a treatment with clozapine or olanzapine not only have a remarkably higher risk for metabolic dysfunction but also are more prone to future adverse cardiovascular outcomes [134]. Furthermore, women taking antipsychotics are 3.6 times more likely to have a prolonged QTc interval than men, with gender predicting QTc length during treatment more than class or type of antipsychotic and age. Women are also at a higher risk of becoming hyperprolactinemic than men while being treated with second generation antipsychotics that block dopamine (risperidone, amisulpride) [138]. That means that, despite the fact that fewer females report sexual dysfunction whilst taking antipsychotics, women are more at risk of developing sexual side effects than men. For what is more, reduced bone density can be associated with low estrogen levels due to chronic elevation of prolactin levels [139]. Thus, ADRs such as amenorrhea, galactorrhea, and sexual dysfunctions might reduce females’ therapeutic adherence to atypical antipsychotics that interfere with the dopamine pathway [140]. On the other hand, antipsychotics that have α1-antiadrenergic and anticholinergic properties are associated with sexual dysfunctions in male patients [141,142,143]. Moreover, acute dystonic reactions, tardive dystonia, and akathisia are more likely to occur in young men than in women [122].

Taking all of the above into account, women appear to have a superior response to antipsychotic therapy, although there are also studies claiming the opposite. However, it seems that reported sex-related differences regarding ADRs are steadier: women are more at risk of developing metabolic dysfunctions, cardiovascular disorders, and hyperprolactinemia. Sexual dysfunctions are common in both sexes, but the underlying mechanism varies in each pharmacological subclass and generates different symptoms.

## 7. Conclusions

So far, many animal and human studies have neglected female subjects in order to avoid a hypothesized complexity of feminine hormonal status. The general assumption was that women possess more variability due to changes during the estrus cycle, thus adding a supplementary layer of complexity to the analyses. As a consequence, studies would require a much larger number of subjects in order to reach adequate power. Nowadays, women may be increasingly represented in clinical trials, but, despite that, most studies fail to analyze sex-related differences in results.

Healthcare professionals need to understand the pharmacokinetics and pharmacodynamics of drugs in various populations thoroughly in order to minimize the ADRs and maximize the therapeutic effectiveness. For this to happen, both sexes should be included in medication trials, clinical, preclinical, and experimental studies in sufficient numbers to detect statistically significant differences. Another step forward would imply that medical trials would include a mandatory report of outcomes differentiated by sex.
